# *Eucrate liui* sp. nov. (Crustacea, Brachyura, Euryplacidae), a new crab species from the South China Sea, with the first record of *Eucrate tripunctata* in the region

**DOI:** 10.3897/zookeys.1284.193896

**Published:** 2026-07-06

**Authors:** Yuan Ziming, Sha Zhongli, Cheng Jiao, Jiang Wei

**Affiliations:** 1 Laboratory of Marine Organism Taxonomy and Phylogeny, Shandong Province Key Laboratory of Marine Biodiversity and Bio-resource Sustainable Utilization, Institute of Oceanology, Chinese Academy of Sciences, Qingdao, China Laboratory of Marine Organism Taxonomy and Phylogeny, Shandong Province Key Laboratory of Marine Biodiversity and Bio-resource Sustainable Utilization, Institute of Oceanology, Chinese Academy of Sciences Qingdao China; 2 Laboratory for Marine Biology and Biotechnology, Qingdao Marine Science and Technology Center, Qingdao, China Marine Biological Museum, Institute of Oceanology, Chinese Academy of Sciences Qingdao China; 3 Marine Biological Museum, Institute of Oceanology, Chinese Academy of Sciences, Qingdao, China Laboratory for Marine Biology and Biotechnology, Qingdao Marine Science and Technology Center Qingdao China

**Keywords:** COI, identification key, morphology, new species, taxonomy

## Abstract

*Eucrate* de Haan, 1835 represents a relatively common goneplacoid genus occurring in benthic habitats of the Indo-West Pacific region, comprising eight recognized species. In the present study, we describe a new species from the South China Sea and report *Eucrate tripunctata* Campbell, 1969 in China for the first time. *Eucrate liui***sp. nov**. bears a close resemblance to *E. tripunctata* but can be distinguished by various carapace features and gonopod morphology. A molecular analysis of the new species based on mitochondrial cytochrome oxidase I sequences supports the recognition of the new species. An updated key to *Eucrate* is also provided.

## Introduction

The family Euryplacidae Stimpson, 1871 was traditionally regarded as a subfamily of Goneplacidae MacLeay, 1838 (Davie 2002; Karasawa and Kato 2003a, 2003b; Castro 2007; Castro and Ng 2010). Based on morphological evidence provided by Guinot (1969), Udekem d’Acoz (1999) elevated it to family status (see also Števčić 2005; Karasawa and Schweitzer 2006; Castro 2007; Ng et al. 2008). The systematics of Euryplacidae was revised by Castro and Ng (2010), who recognized 14 genera. More recently, Ng et al. (2019) transferred *Nectopanope* Wood-Mason in Wood-Mason & Alcock, 1891, a genus previously placed in Xanthidae, to Euryplacidae based on the morphology of the male pleon and gonopods. Consequently, Euryplacidae now comprises 15 known genera.

*Eucrate* de Haan, 1835 is one of the most species-rich genera within Euryplacidae and currently includes eight recognized species: *E. alcocki* Serène, 1973, *E. crenata* (de Haan, 1835), *E. dorsalis* (White, 1849), *E. formosensis* Sakai, 1974, *E. indica* Castro & Ng, 2010, *E. sexdentata* Haswell, 1881, *E. solaris* Yang & Sun, 1979, and *E. tripunctata* Campbell, 1969. These species are predominantly distributed in the Indo-West Pacific region and inhabit subtidal zones ranging from shallow water to depths of at least 200 m (Castro and Ng 2010). Among them, four species, namely *E. alcocki*, *E. crenata*, *E. formosensis*, and *E. solaris*, have been reported from China (Liu 2008; Castro and Ng 2010; Ng et al. 2017). The genus is characterized by a narrow, slender, T-shaped male pleon; a trapezoidal carapace with the anterolateral margins bearing two or three subdued teeth; eyestalks that are either as long as or only slightly longer than their spherical corneas; a proportionally slender propodus and dactylus of the fifth pereiopod (P5); and a G1 with an acuminate apex, the distal part of which bears dense denticles on its lateral surfaces (Castro and Ng 2010).

Recently, in our examination of *Eucrate* species from Chinese waters, *E. tripunctata* is reported for the first time from China. Among the material collected from the South China Sea, some individuals exhibit unique morphological features that distinguish them from the closely related *E. tripunctata*. Careful morphological observations and molecular analyses confirm that they represent a distinct new species. Molecular analysis based on mitochondrial cytochrome oxidase I sequences further corroborates the relationship between the new species and its congeners. We provide a description of this species, along with an updated key to all known *Eucrate* species. The type series of the new species is deposited in the Marine Biological Museum, Chinese Academy of Sciences.

## Materials and methods

Morphological terminology mainly followed that of Castro (2007) and Castro and Ng (2010). The following abbreviations were used in the text: **G1**: first gonopod of male; **G2**: second gonopod of male; **st 1–8**: thoracic sternites 1–8; **pl 1–6**: pleonites 1–6; **P 1–5**: pereopods 1–5.

The specimens are kept in 70% ethanol and deposited in the Marine Biological Museum, Chinese Academy of Sciences (**MBMCAS**), Qingdao, China. Morphological observations were made using a ZEISS Stemi 2000-c microscope, a Zeiss Stemi SV 11 Apo stereo microscope and a Nikon Eclipse Ci-L microscope. Photographs were taken by a Nikon D800 camera with a Nikon AF-S 105 mm lens. The first and second pairs of male gonopods were studied using a Hitachi S-3400N scanning electron microscope (SEM).

Genomic DNA of crabs was extracted from muscle tissue using the Omega E.Z.N.A. Tissue DNA Kit. Mitochondrial cytochrome oxidase I (COI, 658 bp) sequences were obtained by polymerase chain reaction (PCR) amplification using the primers jgLCO1490 and jgHCO2198 (Geller et al. 2013) for molecular phylogenetic analyses. PCR was performed in 25 μl volumes containing: 1 μl (3–200 ng) of genomic DNA template, 1 μl (10 μM) of each primer, 12 μl of 2× PCR Mix (Dongsheng Biotech, Guangzhou, China), and 10 μl of ultrapure water. Reactions were carried out with initial denaturation at 94 °C for 3 min; 35 cycles for denaturation at 94 °C for 30 s, annealing at 48 °C for 45 s, extension at 72 °C for 45 s, and final extension at 72 °C for 10 min.

The nucleotide sequences were aligned using MUSCLE default settings in MEGA v. 6.06 (Tamura et al. 2013). The phylogenetic trees were reconstructed using neighbour-joining (NJ) and Bayesian inference (BI) algorithms as implemented in MEGA v. 6.06 and MrBayes v. 3.2.7 (Huelsenbeck and Ronquist 2001), with *Eriphia smithii* MacLeay, 1838 (Eriphiidae) and *Menippe rumphii* (Fabricius, 1798) (Menippidae) selected as outgroups (Table 1). The best-fitting model was selected using jModelTest v. 0.1.1 under the Akaike information criterion (AIC) (Posada 2008). The sequence divergences of COI between and within species were calculated using a Kimura 2-parameter distance model in MEGA v. 6.06.

**Table 1.** Species and sequences used in the phylogenetic analysis, with GenBank accession numbers.

Species	Location	Voucher ID	GenBank accession number
*Eucrate liui* sp. nov.	Hengling Village, Weizhou Island, Beihai, Guangxi	MBM283322	OL960458
*Eucrate liui* sp. nov.	Daoliao Village, Xinying, Hainan	MBM283320	OL960447
*Eucrate liui* sp. nov.	Linchang reef, Hainan	MBM283331	OL960455
*Eucrate tripunctata* Campbell, 1969	Dijiao wharf, Beihai, Guangxi	MBM283307	OL960454
*Eucrate tripunctata* Campbell, 1969	Dijiao wharf, Beihai, Guangxi	MBM283306	OL960456
*Eucrate tripunctata* Campbell, 1969	Dijiao wharf, Beihai, Guangxi	MBM283301	OL960457
*Eucrate tripunctata* Campbell, 1969	Dijiao wharf, Beihai, Guangxi	MBM283303	OL960446
*Eucrate crenata* (de Haan, 1835)	Qingdao, Shandong	MBM287001	OL960449
*Eucrate crenata* (de Haan, 1835)	Dijiao wharf, Beihai, Guangxi	MBM283302	OL960448
*Eucrate crenata* (de Haan, 1835)	the Yellow Sea	MBM281546	OL960450
*Eucrate crenata* (de Haan, 1835)	Dijiao wharf, Beihai, Guangxi	MBM283312	OL960451
*Eriphia smithii* MacLeay, 1838	north of Qingaowan, Shantou, Guangdong	135CC06688	OL960452
*Menippe rumphii* (Fabricius, 1798)	Weizhou Island, Guangxi	135CC06697	OL960453

## Systematic account

### Family Euryplacidae Stimpson, 1871

**Genus *Eucrate* de Haan, 1835**

#### Eucrate liui sp. nov.

Taxon classificationAnimaliaDecapodaEuryplacidae

6EB85DCB-9EE1-5D9E-9473-652B0DA9D11E

https://zoobank.org/32DC0CA8-DFF3-4F71-B3F3-074E692AF499

**Figure 1.**
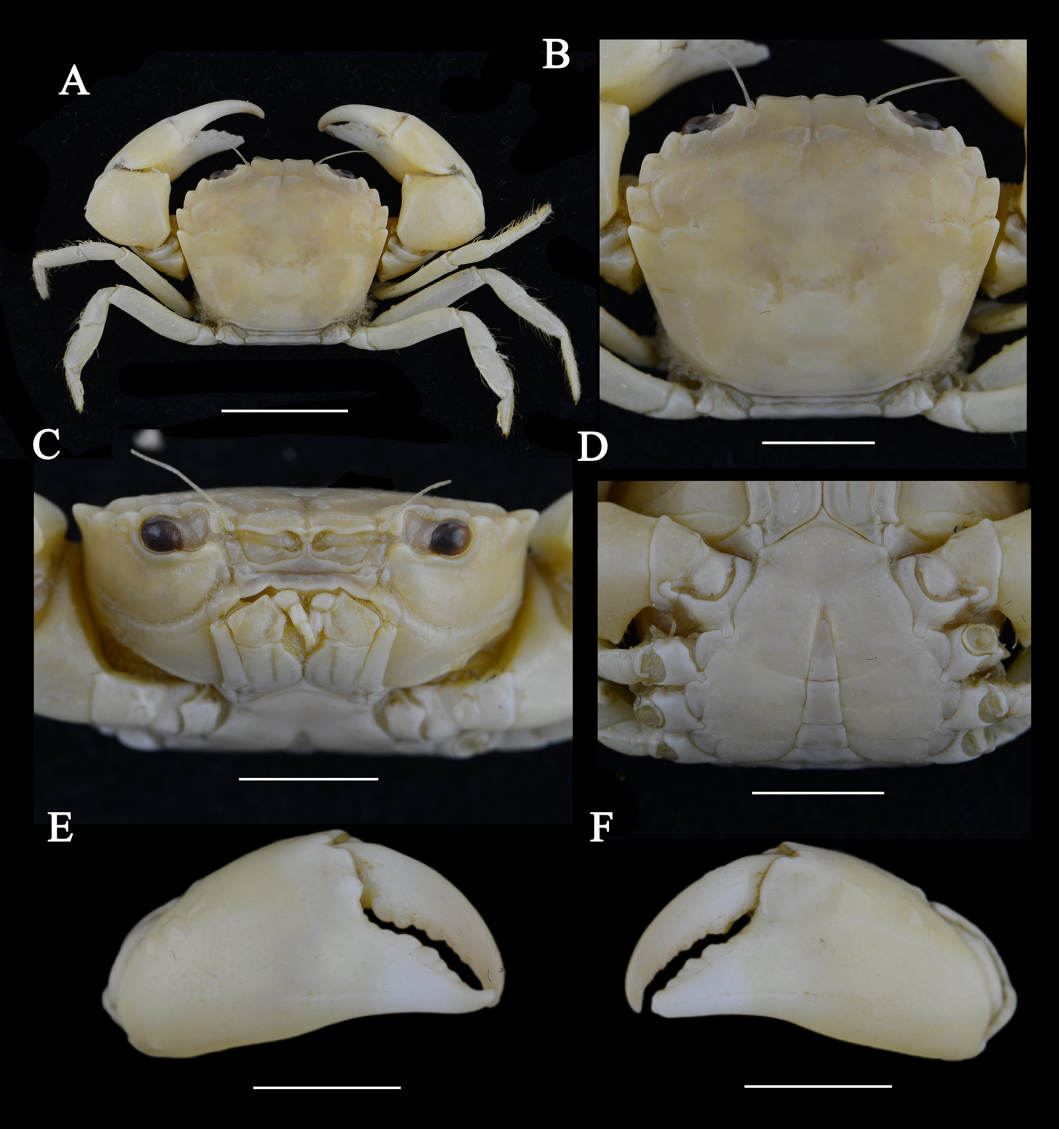
*Eucrate liui* sp. nov., male holotype (16.46 × 13.47 mm) (MBM167054). **A**. Overall body; **B**. Carapace; **C**. Frontal view; **D**. Thoracic sternites and abdomen; **E**. Right cheliped; **F**. Left cheliped. Scale bars: 10 mm (**A**); 5 mm (**B–F**).

**Figure 2.**
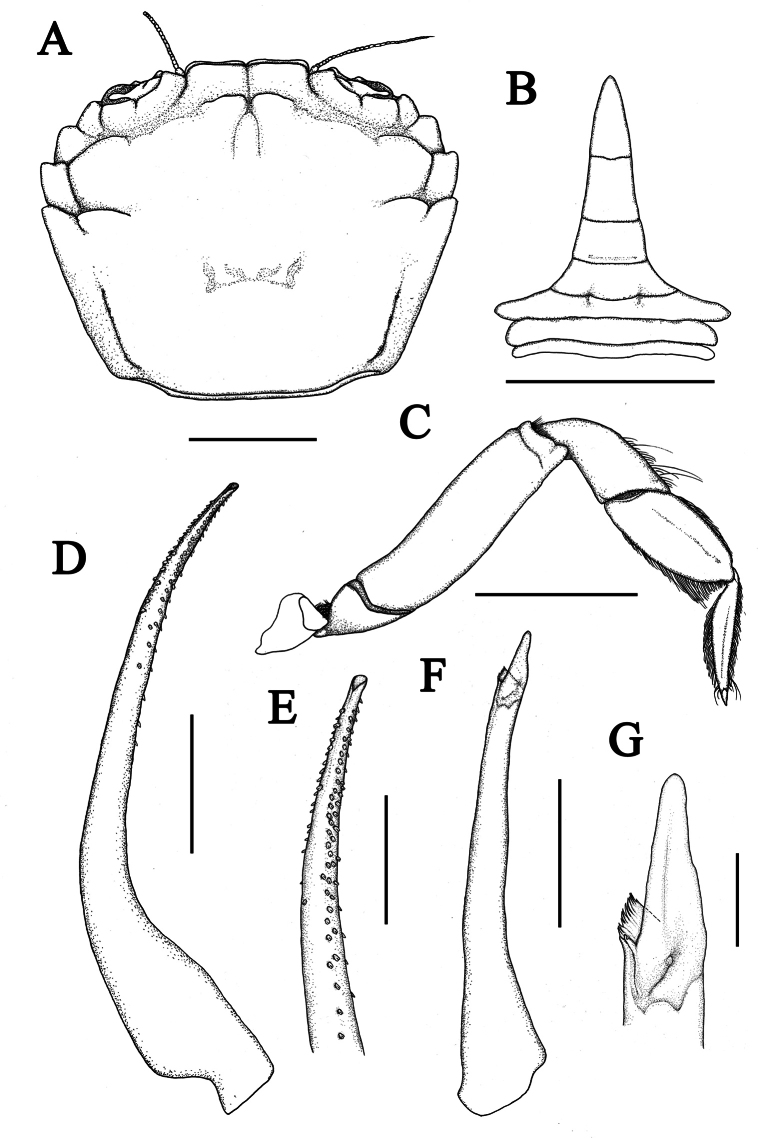
*Eucrate liui* sp. nov., male holotype (16.46 × 13.47 mm) (MBM167054). **A**. Carapace; **B**. Abdomen; **C**. P5, right; **D**. G1, right, ventral view; **E**. Same, distal part; **F**. G2, right, ventral view; **G**. Same, distal part. Scale bars: 5 mm (**A–C**); 1 mm (**D**); 0.5 mm (**E, F**); 0.1 mm (**G**).

**Figure 3.**
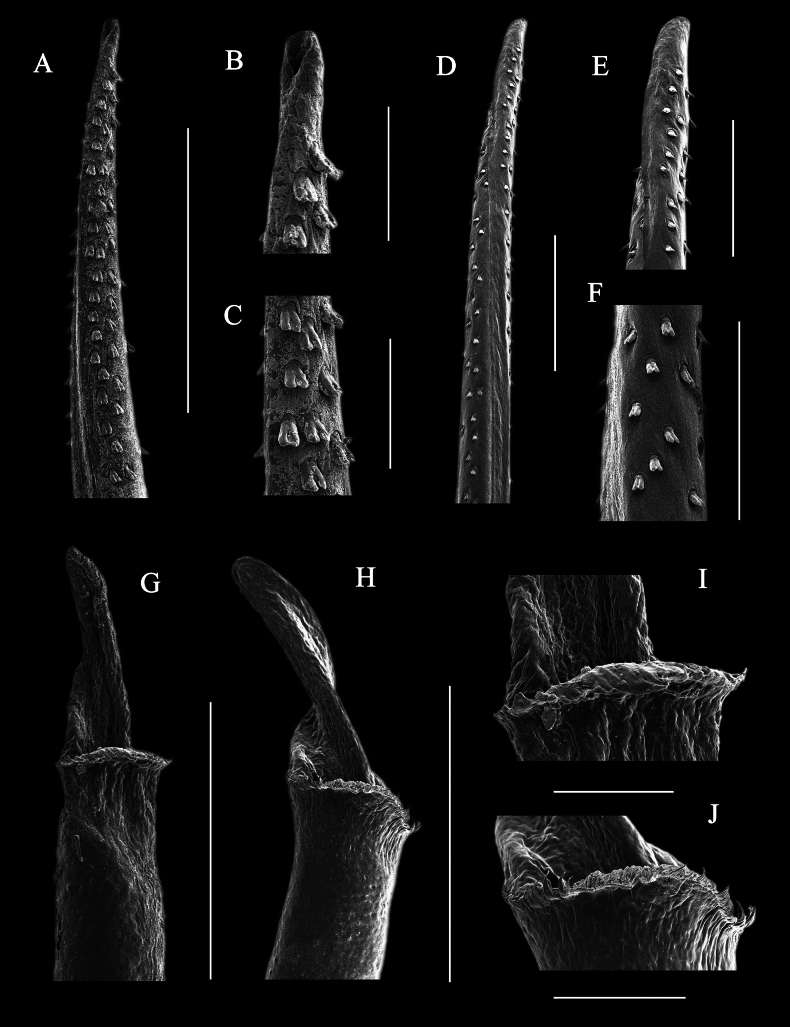
SEM of the male gonopods. **A**. G1 of *E. liui* sp. nov., distal part, ventral view; **B**. Same, showing the tip; **C**. Same, showing the bifid squamous spines; **D**. G1 of *E. tripunctata*, distal part, ventral view; **E**. Same, showing the tip; **F**. Same, showing bifid squamous spines; **G**. G2 of *E. liui* sp. nov., distal part; **H**. G2 of *E. tripunctata*, distal part; **I**. G2 subdistal lobe of *E. liui* sp. nov.; **J**. G2 subdistal lobe of *E. tripunctata*.

**Figure 4.**
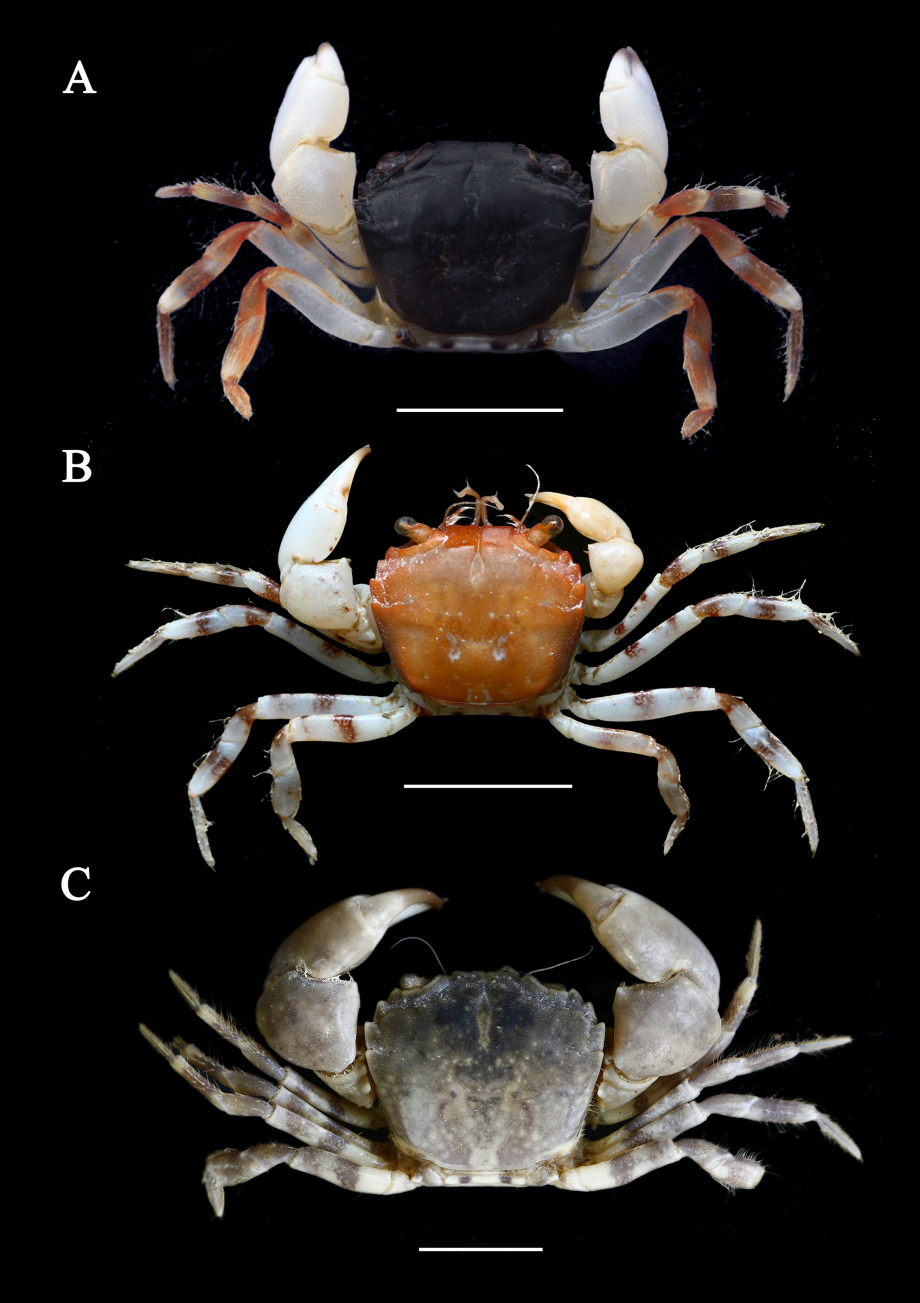
*Eucrate liui* sp. nov., live colouration. **A**. Male (7.10 × 5.80 mm) (MBM283320); **B**. Female (12.06 × 10.20 mm) (MBM283331); **C**. Male (19.30 × 16.10 mm) (MBM283322). Scale bars: 5 mm (**A**); 10 mm (**B, C**).

**Figure 5.**
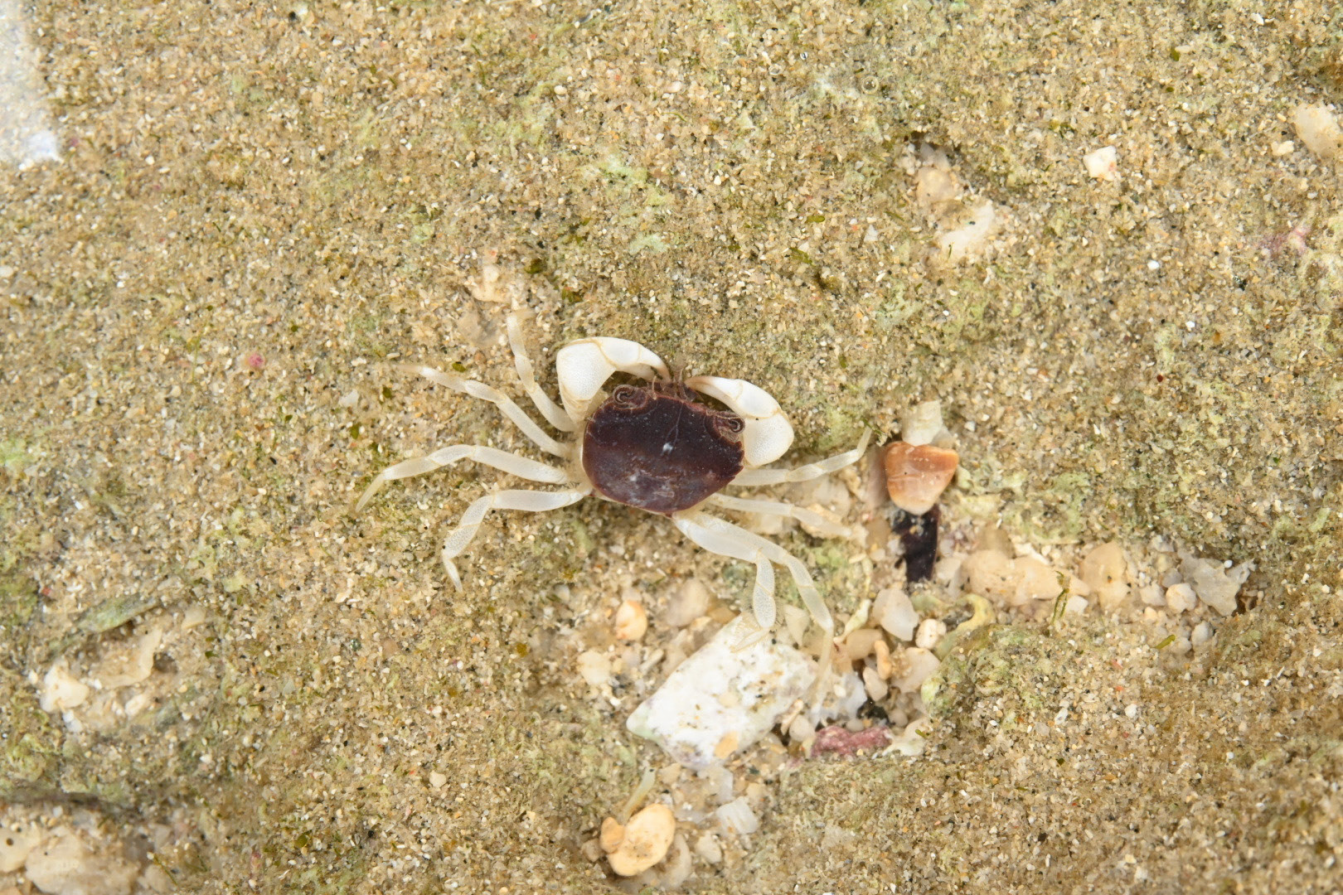
*Eucrate liui* sp. nov., live specimen *in situ*, photographed at Wenchang, Hainan, by Zhang Xu.

**Figure 6.**
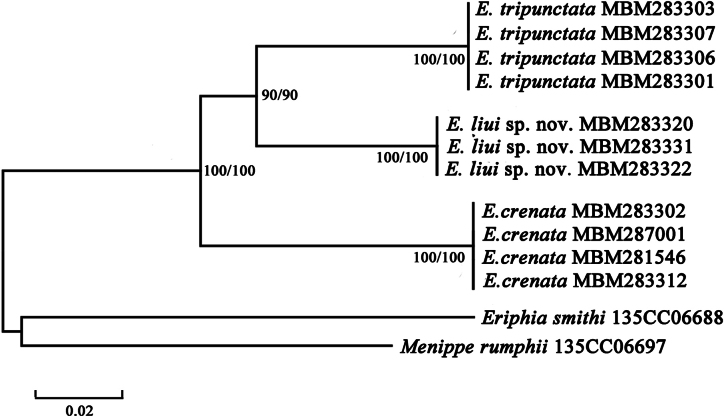
Phylogenetic relationships among *E. liui* sp. nov. and other two *Eucrate* species based on COI sequences. The NJ tree was shown, with NJ bootstrap replications and BI posterior probabilities labeled around branches (BS/PP).

Figs 1, 2, 3, 4, 5, 6

Eucrate tripunctata —Castro and Ng 2010: 40–45, fig. 12D–F (in part). Non Eucrate tripunctataCampbell 1969.

##### Material examined.

***Holotype***: China • 1 ♂, 16.5 × 13.5 mm; Aotou, Huizhou, Guangdong; 30 May 1976; leg. Sun Ruiping; MBM167054.

***Paratypes***: China • 1 ♂, 19.3 × 16.1 mm, Hengling Village, Weizhou Island, Beihai, Guangxi; 25 Apr. 2010; leg. Wang Haiyan et al; low tide zone; MBM283322 • 1 ♂, 7.1 × 5.8 mm; Daoliao Village, Xinying, Hainan; 27 Nov. 2007; leg. Wang Yongliang; under rubble, green algae nearby, intertidal zone; MBM283320 • 1 ♀, 12.1 × 10.2 mm; Linchang reef, Hainan; 12 Apr. 2008; leg. Wang Yongliang & Li Changwen; under rubble, coral reef; MBM283331 • 1 ♀, 12.1 × 9.8 mm, Linchang reef, Hainan; 11 Feb. 1992; MBM167049.

##### Comparative material.

*Eucrate tripunctata* Campbell, 1969.

See the material examined of *E. tripunctata*.

*Eucrate crenata* (de Haan, 1835).

China • 1 ♂, 1 ♀; Dijiao wharf, Beihai, Guangxi; 11 Apr. 2010; leg. Jiang Wei; MBM283302 • 1 ♂, 3 ♀; Yellow Sea; 15 Jul. 2010, leg. Jiang Wei et al.; MBM281546 • 3 ♂; Dijiao wharf, Beihai, Guangxi; 11 Apr. 2010; leg. Jiang Wei; MBM283312 • 1 ♀; Qingdao, Shandong; 3 Nov. 2019; leg. Yuan Ziming & Meng Fei; MBM287001.

##### Diagnosis.

Carapace smooth; dorsal surface flat, with distinct ridges behind front, before second and third anterolateral teeth, and parallel to posterolateral margin; anterior margin with three triangular lobes; outer orbital teeth triangular and prominent; front prominent, divided into two lobes by a deep notch, followed by a shallow groove, which bifurcates at anterior margin of mesogastric region; frontal lobes concave. Pereopod 5 propodus and dactylus flat. Male pleon slender, T-shaped, lateral margins of pl 6 straight. First gonopod of male slender, curving laterally, with bifid squamous denticles. Second gonopod of male small, slightly curving, subdistal with subtriangular lobe.

##### Description.

***Carapace*** (Figs 1A, 1B, 2A) trapezoidal, breadth about 1.22 times length; dorsal surface smooth, flat, with anterior slightly elevated, posterior flat, and regions indistinct; front divided into two lobes by a median notch; front lobes slightly concave, with shallow groove originating from frontal notch, bifurcating at anterior margin of mesogastric region; orbit shorter than front, separated from front by deep notch; dorsal orbital edge with double folds, ventral orbital edge sinuous, middle lobe obtusely triangular, ventral inner orbital angle slightly prominent, blunt. Eye peduncles short, subequal to cornea; cornea large, spherical. Antennal basis disto-laterally prolonged, filling orbital hiatus; antennal flagellum excluded from orbit, long, reaching second anterolateral tooth. Outer orbital teeth triangular; anterolateral border armed with three triangular teeth excluding outer orbital teeth; first tooth broad, with obtusely angled tip; second most prominent, with acutely angled tip; third similar to second but less prominent and posterior margin extending backward. Posterolateral margin slightly converging backwards, longer than anterolateral margin. Carapace dorsal surface with four distinct pairs of ridges: one pair behind front, parallel to front; two pairs of curved smooth transverse ridges before second and third anterolateral teeth, parallel to each other; one pair of long ridges with irregular fine granules, parallel to posterolateral margin. Shallow H-shaped groove present between gastric and cardiac regions.

***Epistome*** (Fig. 1C) with lobular posterior margin and lateral lobes sinuous, divided from median lobes by a fissure; median portion with two lobes divided by a deep fissure. Third maxillipeds (Fig. 1C) completely covering buccal space; ischium subrectangular, with submedian longitudinal groove; merus subpentagonal, with anterolateral angle produced.

***Thoracic sternum*** (Fig. 1D) smooth, st 1, 2 fused, suture between st2, 3 distinct, curved forward; suture between st 3 and 4 only present on lateral edges; st 4 broad; sterno-pleonal lock located on posterior of st 5; st 8 completely covered by abdomen in male.

***Chelipeds*** (Fig. 1E, F) nearly equal, right slightly larger than left, robust; merus armed with two tubercles and strong subterminal teeth; carpus with internal tooth, and inner anterior margin with short tomentum; palm smooth, with outer surface and carpus covered by similar short tomentum; shallow groove present close to ventral margin, parallel to ventral margin; dactylus longer than dorsal margin of palm; fingers with sharp tips, cutting edges with blunt teeth; base of dactylus with tuft of tomentum.

***Ambulatory legs*** (Figs 1A, 2C) slender, smooth; margins of merus, propodus, and dactylus with setae; anterior margin of carpus with setae; dactylus slender, sharp; P4 longest, P3 and P5 shortest, similar. P5 (Fig. 2C) merus sinuous, nearly reaching third anterolateral tooth when folded; propodus and dactylus flat, propodus short, wide, about twice as long as wide.

***Male pleon*** (Figs 1D, 2B) slender, T-shaped; pl 1–3 wide, abruptly narrowing from pl 4; telson slender, tip sharp.

G1 (Figs 2D, 2E, 3A–C) slender, curving laterally, with bifid squamous denticles on distal third, tip opening ventral, teardrop-shaped. G2 (Figs 2F, 2G, 3G, 3I) small, slightly curving, subdistal, with subtriangular lobe; margin of subdistal lobe divided into sharp, narrow setaceous lobes.

##### Live colouration.

This species shows significant variation in body colouration, which can be broadly divided into two types (Fig. 4). Type A (Figs 4A, 4B, 5): carapace orange-red to nearly black or dark violet; chelipeds white; ambulatory legs white, with orange-red rings. Type B (Fig. 4C): carapace grey to brown, darker in gastric region, with white marbling; chelipeds white, with grey reticulation; ambulatory legs white, with grey rings. These colour variations may be attributed to individual growth stages or environmental factors within the habitat.

##### Distribution.

The present specimens were collected from Guangdong (type locality), Guangxi, and Hainan, South China Sea, and were living under rubble in the intertidal zone with green algae and coral reefs. The species is also known from Singapore (Castro and Ng 2010).

##### Etymology.

This species is named after the late Prof. Liu Ruiyu (J.Y. Liu), the Institute of Oceanology, Chinese Academy of Sciences, for his significant contributions to Chinese marine science.

##### Remarks.

**Figure 7.**
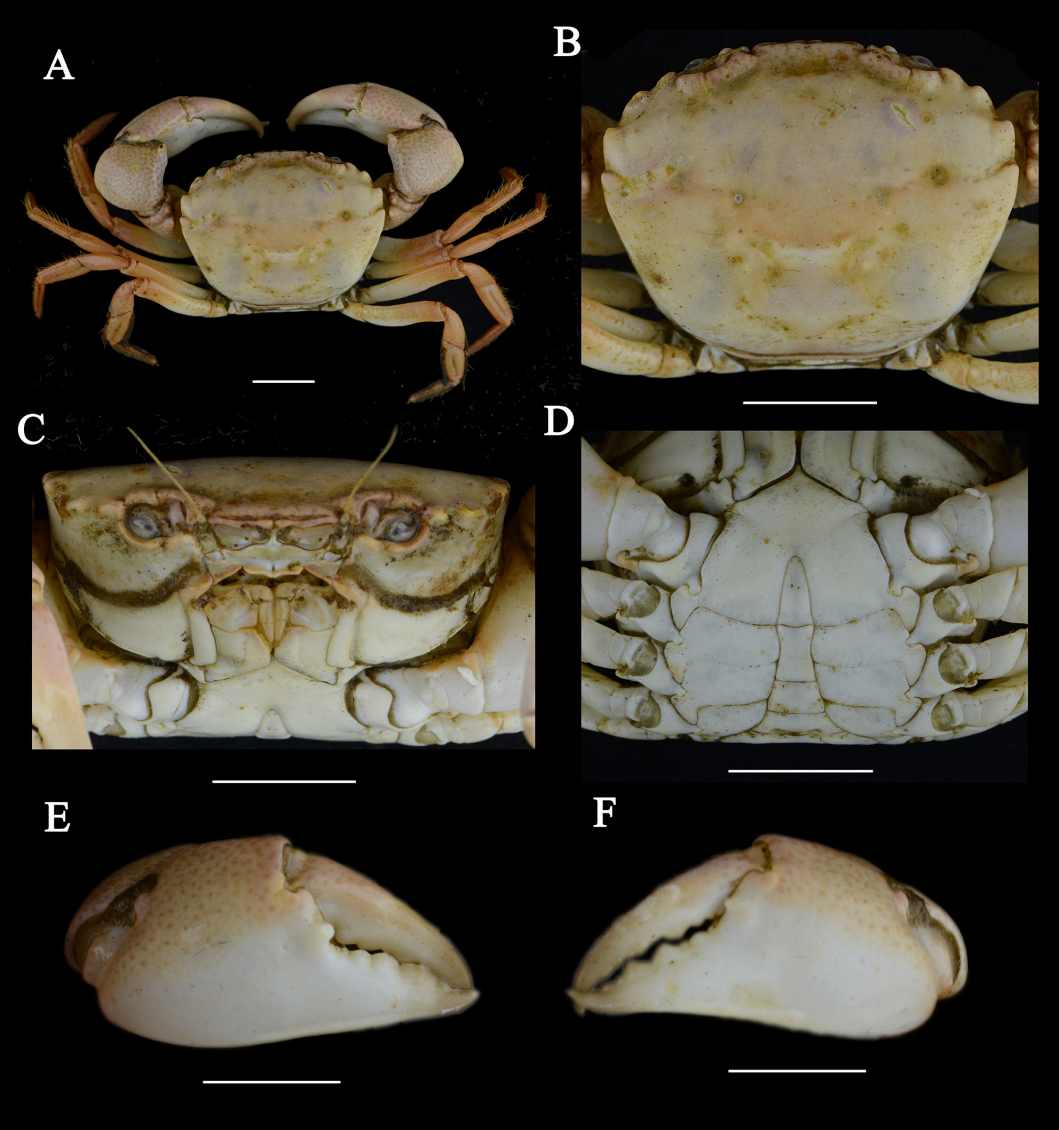
*Eucrate tripunctata*, male (31.29 × 25.03 mm) (MBM287002). **A**. Overall; **B**. Carapace; **C**. Frontal view; **D**. Thoracic sternites and abdomen; **E**. Right cheliped; **F**. Left cheliped. Scale bars: 10 mm.

**Figure 8.**
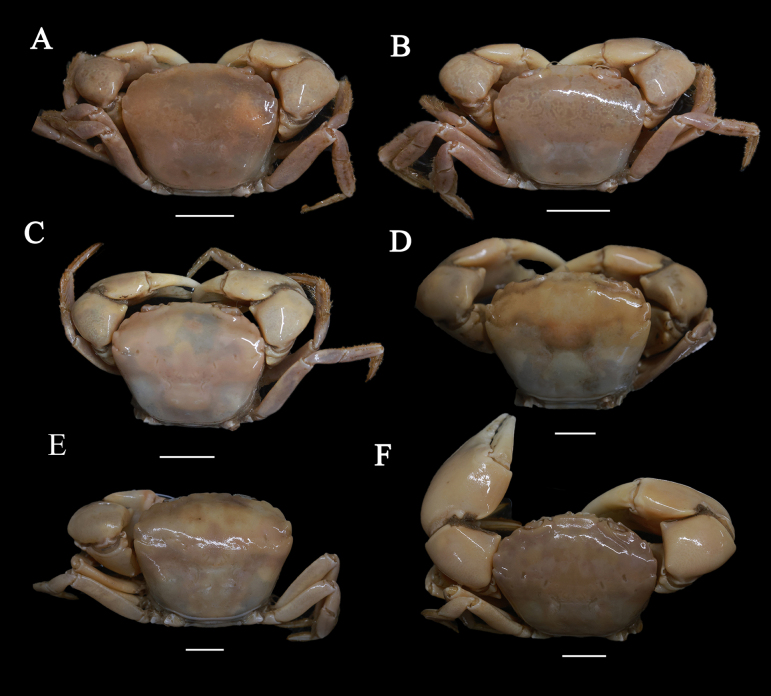
*Eucrate tripunctata*, holotype, male (34.4 × 25.6 mm) (W3034). **A**. Overall; **B**. Carapace; **C**. Frontal view; **D**. Thoracic sternites and abdomen; **E**. Right cheliped; **F**. Left cheliped. Scale bars: 10 mm.

**Figure 9.**
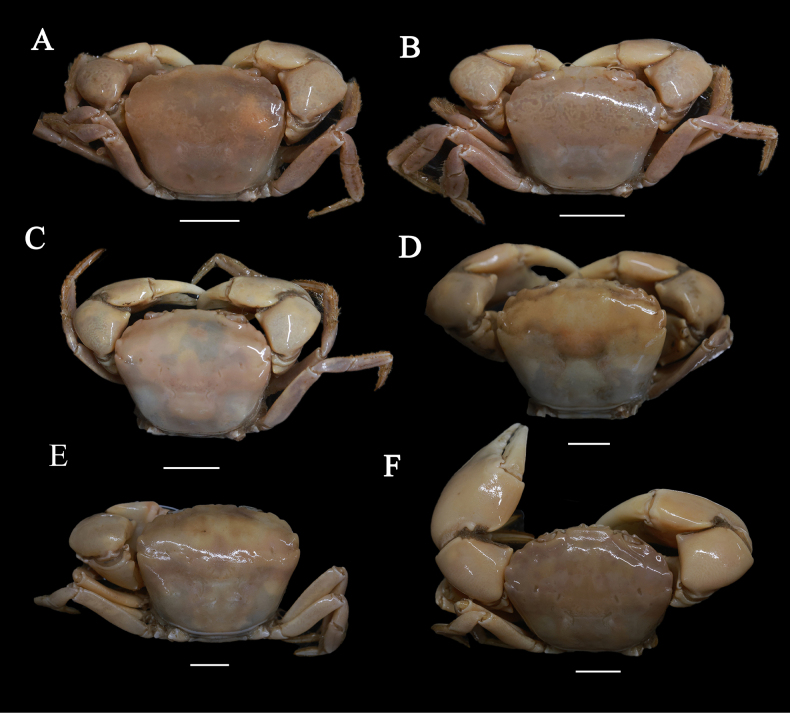
*Eucrate tripunctata*, paratypes. **A–C**. Females (26.0–29.7 × 23.8–20.6 mm) (W3034); **D**. Male (33.6 × 21.2 mm) (W1070); **E**. Female (46.4 × 37.6 mm) (W1190); **F**. male (34.6 × 25.1 mm) (W1496).

**Figure 10.**
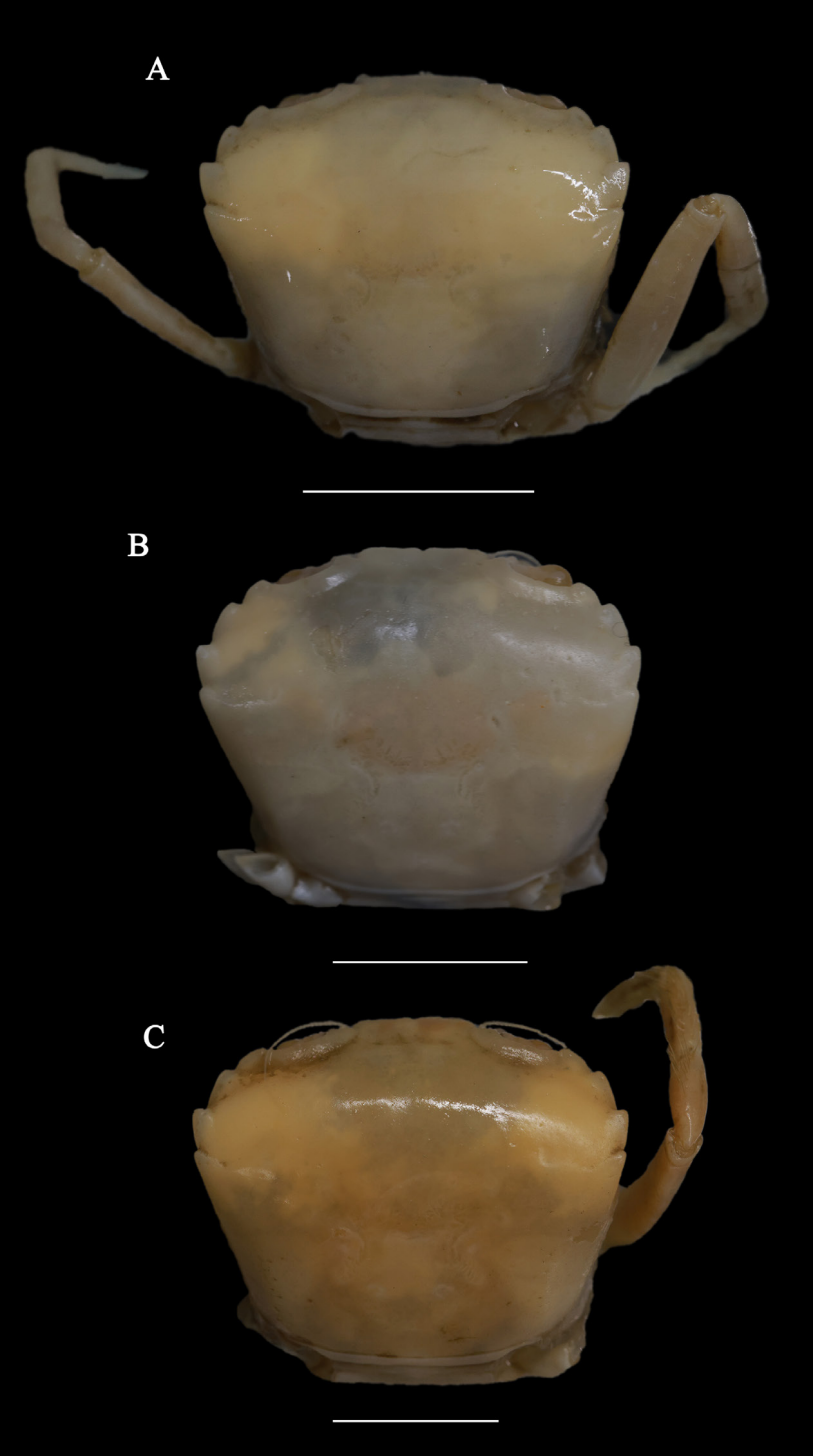
*Eucrate tripunctata*, paratypes. **A**. Female (19.8 × 15.8 mm) (W3029); **B**. Female (24.8 × 18.4 mm) (W1541); **C**. Female (24.87 × 19.1 mm) (W3030).

*Eucrate liui* sp. nov. is closely related to *E. tripunctata* in morphology but shows distinct differences in the following: carapace dorsal surface flat, with anterior only slightly elevated (Figs 1B, 2A) (dorsal surface arched and anterior part more elevated in *E. tripunctata*; Figs 7B, 8B, 9, 10); anterolateral teeth more prominent and sharp, with outer orbital tooth triangular and well developed (Figs 1B, 2A) (anterolateral tooth not so prominent, blunter, the outer orbital teeth low and inclined forward, with outer margin round in *E. tripunctata*; Figs 7B, 8B, 9, 10); front more prominent, divided into two lobes by a distinct middle notch, with a shallow bifurcating groove at anterior margin of mesogastric region and frontal lobes concave (Figs 1B, 2A) (front not prominent, only with a slight indentation at middle, without a groove, and frontal lobe margin nearly straight in *E. tripunctata*; Figs 7B, 8B, 9, 10); carapace with distinct ridges present behind front, before second and third anterolateral teeth, and parallel to posterolateral margin (Figs 1B, 2A) (carapace without ridges, or only with very faint ridges behind front and before 3^rd^ anterolateral teeth on small specimens in *E. tripunctata*; Figs 7B, 8B, 9, 10); telson and pl 6 wider, lateral margin of pl 6 nearly straight (Figs 1D, 2B) (telson and pl 6 slender, lateral margin of pl 6 concave in *E. tripunctata*; Figs 7D, 8D). Furthermore, the two species exhibit distinct differences in body colouration (Figs 4, 5, 10). Subtle variations are also discernible in the G1 structures between the two species: in *E. liui*, the G1 is shorter with a relatively uniform thickness, features a larger, ventrally oriented, teardrop-shaped tip opening, and has proportionally larger denticles (Figs 2D, 2E, 3A–C). This stands in contrast to the G1 structure in *E. tripunctata*, which is slender, narrows abruptly on its basal third, has a smaller, upward-oriented tip opening that is not visible ventrally, and possesses proportionally smaller denticles (Fig. 3D–F).

Considering that the present specimens are consistently smaller than those of *E. tripunctata*, size-matched comparisons are important. Fortunately, within the type series of *E. tripunctata*, we were able to locate three smaller female individuals that are only marginally larger than the new species. Although they exhibit more pronounced and sharper anterolateral teeth than larger individuals, these smaller specimens can still be differentiated from the new species by their frontal characteristics, the absence of a groove, low-lying outer orbital teeth, dorsal convexity, and the indistinct carapace ridges.

Based on the available data, the new species appears to be of a significantly smaller than *E. tripunctata*, making it prone to confusion with the juvenile crabs of the latter. Part of the *E. tripunctata* material from Singapore examined by Castro and Ng (2010) should be referred to the present new species based on their highly similar morphology and living colouration.

*Eucrate liui* sp. nov. is also similar to *E. crenata* (de Haan, 1835), but it can be easily distinguished by the following: three triangular anterolateral teeth, the first slightly broader, the third similar to the second (Figs 1B, 2A) (in *E. crenata*, the first tooth distinctly low and broad, with a wider gap between the second and third teeth, the third tooth smaller than the second); carapace with ridges (Figs 1B, 2A) (carapace without obvious ridges in *E. crenata*); cheliped carpus with short tomentum on inner anterior margin (Fig. 1A) (cheliped carpus with short tomentum on outer anterior margin in *E. crenata*); P5 propodus short and wide (Fig. 2C) (P5 propodus slender in *E. crenata*).

The pattern of phylogenetic relationships among all studied *Eucrate* species was identical across the analytical methods (Fig. 6). All individuals of the same *Eucrate* species grouped into a single cluster with high support values (100/100 for all three species), and *E. liui* sp. nov. was clustered with *E. tripunctata* (90/90), which is in accordance with the morphological investigations. In addition, the COI sequence divergences between the three *Eucrate* species were higher than 8% (Table 2). This divergence level exceeded the COI barcoding threshold applied to alpheid shrimps (Knowlton and Weigt 1998) and mantis shrimps (Barber and Boyce 2006). Both morphological and molecular evidence support the new species.

**Table 2.** Sequence divergence of COI gene among *E. liui* sp. nov. and other two *Eucrate* species inferred from Kimura 2-parameter-corrected calculations.

	*E. liui* sp. nov.	*E. tripunctata*	*E. crenata*
*E. liui* sp. nov.	0.000		
*E. tripunctata*	0.087	0.000	
*E. crenata*	0.111	0.121	0.000

Considering the existence of some unresolved specimens in the genus *Eucrate*, we discuss the dubious specimens that resemble the new species to facilitate future examination. Campbell (1969) attributed the specimens from the Mergui Archipelago, initially identified as *E. affinis* (a synonym of *Trissoplax dentata* (Stimpson, 1858)) by de Man (1887), to *E. tripunctata*, as well as a single specimen of the same collection identified as *Eucrate crenata* var. affinis by Alcock (1900). However, Castro and Ng (2010) argued that these specimens should not be classified as *E. tripunctata*, primarily due to the sharp anterolateral teeth and shorter telson. The description provided by de Man (1887) is similar to the new species; however, there are minor discrepancies, notably in de Man’s figure, where the carpus appears more inflated. The Pakistan specimens identified as *E. sulcatifrons* (a synonym of *Eucrate crenata* (de Haan, 1835)) by Tirmizi and Ghani (Tirmizi and Ghani 1982, 1996) are also similar to the present species in morphological characteristics and living colouration, except for a minor difference in the G1 structure. In the figures provided by Tirmizi and Ghani (Tirmizi and Ghani 1982, 1996), the apical opening of the G1 is shown as not inclined, in contrast to the present specimens, where this opening is inclined toward the abdomen, forming a teardrop shape. Due to these observed differences, additional scrutiny of this material is advised to accurately determine its taxonomic status.

#### Eucrate tripunctata

Taxon classificationAnimaliaDecapodaEuryplacidae

Campbell, 1969

934DA35C-8ED2-5C99-B70C-D21D10DBBD29

**Figure 11.**
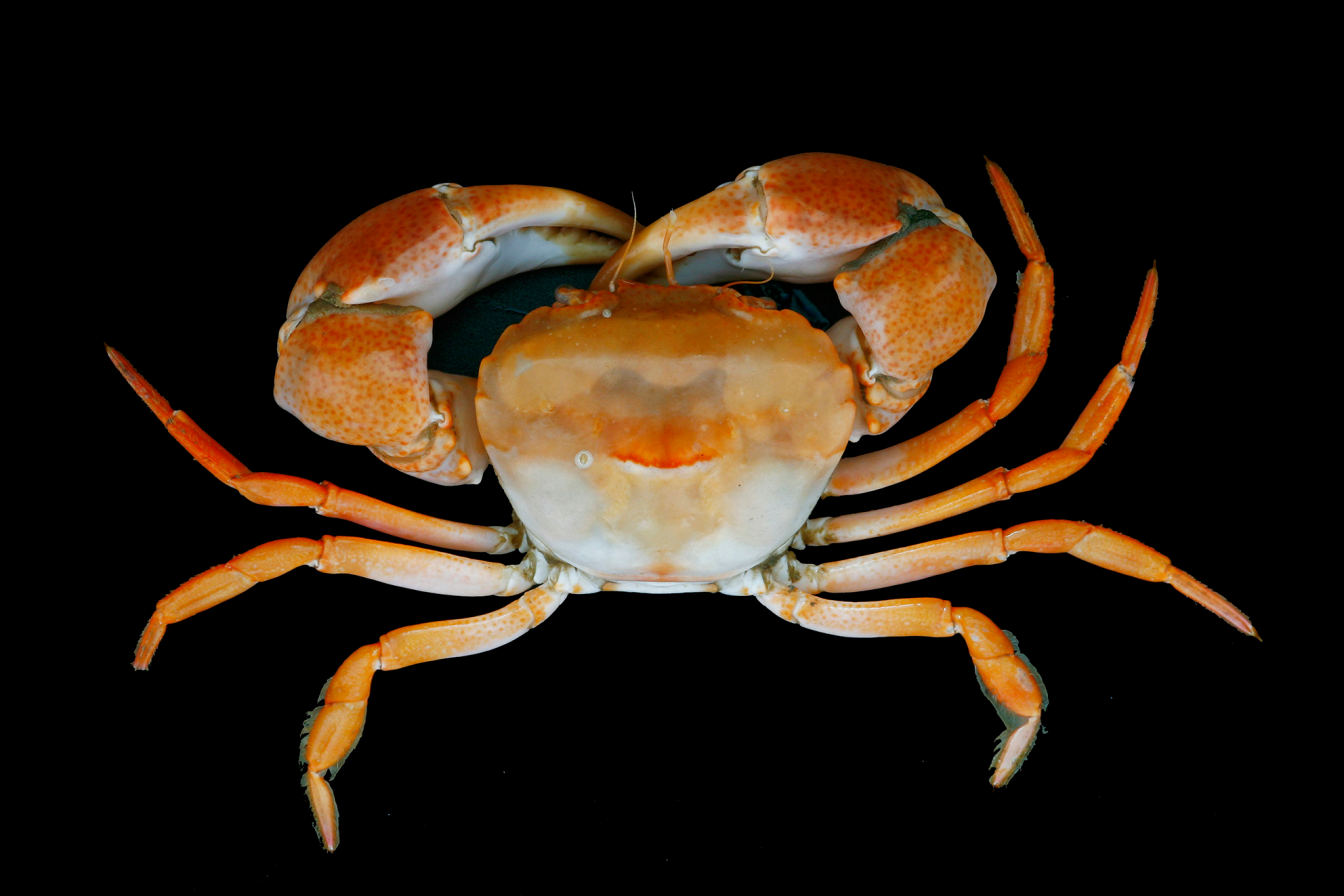
*Eucrate tripunctata*, male (45.47 × 36.00 mm) (MBM283306), show live colouration.

Figs 7, 8, 9, 10, 11

Eucrate tripunctata Campbell 1969: 119 (in key), 127, figs 2, 4—Guinot 1971: 1080 (in list); Davie 2002: 199 (in list); Ng et al. 2008: 78 (in list); Castro and Ng 2010: 40–45, fig. 12A–C (in part).

##### Material examined.

***Holotype***: Australia • 1 ♂, 34.4 × 25.6 mm; Mud I., Moreton Bay, Queensland; 26 May. 1944; leg. V.F. Collin; W3034.

***Paratypes***: Australia • 3 ♀, 26.0–29.7 × 23.8–20.6 mm; Mud I., Moreton Bay, Queensland; W3034 • 1 ♀, 44.0 × 32.4 mm; Mud I., Moreton Bay, Queensland; 5 Jun. 1932; W384 • 1 ♂, 33.6 × 21.2 mm; Mud I., Moreton Bay, Queensland; leg. V.F. Collin; W1070. 1 ♀, 46.4 × 37.6 mm; Mud I., Moreton Bay, Queensland; 15 Jan. 1941; leg. V.F. Collin; W1190 • 1 ♂, 34.6 × 25.1 mm; Mud I., Moreton Bay, Queensland; leg. V.F. Collin; W1496 • 1 ♀, 24.8 × 18.4 mm; Mud I., Moreton Bay, Queensland; W1541. 1 ♀, 19.8 × 15.8 mm; Mud I., Moreton Bay, Queensland; 12 Oct. 1942; leg. V.F. Collin; W3029 • 1 ♀, 24.9 × 19.1 mm; Mud I., Moreton Bay, Queensland; 12 Oct. 1942; leg. V.F. Collin; W3030.

##### Other specimens.

China • 1 ♀, 31.0 × 24.3 mm; Dijiao wharf, Beihai, Guangxi; 11 Apr. 2010, leg. Jiang Wei; MBM283307 • 1 ♂, 45.5 × 36.0 mm; Dijiao wharf, Beihai, Guangxi; 11 Apr. 2010; leg. Jiang Wei; MBM283306 • 1 ♂, 42.7 × 34.2 mm; Dijiao wharf, Beihai, Guangxi; 12 Apr. 2010; leg. Jiang Wei; MBM283301 • 1 ♀, 50.7 × 39.3 mm; Dijiao wharf, Beihai, Guangxi; 11 Apr. 2010; leg. Jiang Wei; MBM283303 • 1 ♂, 31.3 × 25.0 mm; Dapeng, Shenzhen, Guangdong; 6–10 m; Sep. 2021; MBM287002 • 1 ♂, 36.0 × 28.1 mm; Dapeng, Shenzhen, Guangdong; 6–10 m; Sep. 2021; MBM287003.

##### Diagnosis.

Carapace smooth, with dorsal surface arched and anterior part elevated, without distinct groove or ridge; anterior margin with three round lobes; outer orbital teeth low and inclined forward; outer margin round (Figs 7A, 7B, 8A, 8B, 9–11); front not prominent, divided into two lobes by a fine fissure, appearing almost intact on dorsal view, frontal margin straight. P5 propodus and dactylus flat (Figs 7A, 11); male pleon slender, T-shaped, lateral margins of pl 6 concave (Figs 7D, 8D). G1 slender, elongate, curving laterally, straight along median portion, narrowed abruptly at the basal 1/3, with bifid squamous denticles (Fig. 3D–F). G2 small, slightly curving, subdistal with round lobe (Fig. 3H, J). In life, carapace pale cream to orange-red, darker in anterior 2/3; chelipeds with dense orange-red spots; ambulatory legs orange-red, base white (Fig. 11). See Campbell (1969) and Castro and Ng (2010).

##### Distribution.

Guangxi, Guangdong, South China Sea; Gulf of Thailand, Singapore, Queensland (type locality).

##### Remarks.

This species is reported for the first time from Chinese waters. The current material is consistent with the type specimens from Australia and the descriptions of Castro and Ng (2010).

In the original description by Campbell (1969), the species was noted to have three large red spots on the dorsal surface of faded alcohol specimens, consisting of one large oval central spot and two smaller lateral spots. However, these spots have not been observed in either fresh or alcohol-preserved Chinese specimens. The Australian type series is now almost completely faded, with only a few specimens still showing a demarcation line between the anterior and posterior portions of the carapace. Most specimens show no noticeable traces of pigmented spots that match the description (Figs 8, 9, 10). The only exception is a small paratype, W1541 (Fig. 10B), in which three darker colour patches are found above and on both sides of the H-shaped groove in the mesogastric region. In the fresh adult specimens examined in this study (Fig. 11), these colour patches correspond to the following structures: a rectangular colour patch in the center, flanked on either side by roughly circular small colour patches. However, the boundaries of these colour patches in the adults are less distinct, making it difficult to refer to them as “three spots”. Further examination of additional specimens is necessary to definitively establish the colouration characteristics of this species.

### Key to species of *Eucrate* de Haan, 1835

(Adapted from Castro and Ng 2010)

**Table:** 

1	Third (excluding outer orbital tooth) anterolateral tooth absent (only slight elevation may be present), carapace with relatively long posterolateral borders	**2**
–	Third (excluding outer orbital tooth) anterolateral tooth present, even if short, sometimes barely noticeable in large individuals, carapace without relatively long posterolateral borders	**5**
2	Conspicuous frontal notch present; in life, large purple-pink spots on anterior two thirds of dorsal surface of carapace	***E. sexdentata***
–	Frontal notch absent or barely noticeable; in life, variously shaped red-brown spots, dots, or many small dots on anterior half of dorsal surface of carapace	**3**
3	In life, anterior half or most of carapace and chelipeds with small red-brown dots and larger dots in small individuals	***E. formosensis***
–	In life, median portion of carapace with large, irregular dark red-brown spots; small red-brown spots on anterior third of carapace and chelipeds	**4**
4	Median portion of carapace with one large, rounded median spot in life, typically flanked on each side and anteriorly by smaller spots; western Pacific in distribution	***E. alcocki***
–	Median portion of carapace with two large, irregular, red-brown submedian spots in life, typically flanked by two smaller, vertically placed spots; Indian Ocean in distribution	***E. indica***
5	P5 propodus noticeably short, wide; three anterolateral teeth short, with second and third nearly equal	**6**
–	P5 propodus slender, not wide; three anterolateral teeth of varying size, with first much smaller than second	**7**
6	Anterolateral teeth round; carapace dorsal surface without distinct groove or ridge; carapace pale cream to orange-red, darker in anterior two-thirds; in life, chelipeds with dense orange-red spots	***E. tripunctata***
–	Anterolateral teeth triangular; carapace dorsal surface with paired ridges present behind the front, before the second and third anterolateral teeth, and parallel to posterolateral margin; in life, colour variable (Figs 4, 5)	***E. liui* sp. nov**.
7	First anterolateral tooth very short, nearly disappearing in large individuals; in life, typically one large spot on central region of carapace in life	***E. dorsalis***
–	First anterolateral tooth well developed, not noticeably reduced in size; carapace with reticulated pattern of spots or two spots on anterior half in life	**8**
8	Ventral surface of cheliped meri with conspicuous, high tubercles; in life, intricate, reticulated pattern of dots, spots across carapace, chelipeds, ambulatory legs	***E. solaris***
–	Ventral surface of cheliped meri smooth or with short granules; in life, small dots on anterior third of carapace and on chelipeds plus two small, oval, red-brown spots may be present on branchial region of carapace at some distance from each other	***E. crenata***

## Supplementary Material

XML Treatment for Eucrate liui

XML Treatment for Eucrate tripunctata

## Additional information

### Artificial Intelligence (AI) use

The authors accept full responsibility for the content of the manuscript, including the disclosure of any use of AI.

No AI tools were used in the preparation of this manuscript.

### Author ORCIDs

Y. Ziming https://orcid.org/0009-0008-0009-2693

S. Zhongli https://orcid.org/0000-0002-2192-3758

C. Jiao https://orcid.org/0000-0003-0820-7762

J. Wei https://orcid.org/0000-0003-4767-8196

### Data availability

All of the data that support the findings of this study are available in the main text.
